# The urgent need for African research collaboration on medicine quality

**DOI:** 10.1038/s41467-025-67430-z

**Published:** 2026-01-10

**Authors:** Fanqi Zeng, Simon Mariwah, Gerry Mshana, Daniel Amoako-Sakyi, Heather Hamill

**Affiliations:** 1 Department of Sociology, University of Oxford, Oxford, UK; 2 Department of Geography and Regional Planning, University of Cape Coast, Cape Coast, Ghana; 3 National Institute of Medical Research, Mwanza, Tanzania; 4 Department of Microbiology and Immunology, University of Cape Coast, Cape Coast, Ghana

**Keywords:** Drug regulation, Developing world, Society, Public health, Health policy

## Abstract

Substandard and falsified medicines are a global health threat. The fight against them is a regulatory and research challenge; here, the authors argue the importance of global and regional oversight, monitoring of, and research into the extent of the issue.

## Teaser

Substandard and falsified medicines threaten African health systems, yet research on this crisis is fragmented and concentrated in few countries, predominantly led by Global North institutions. Building African-led research capacity is essential for evidence-based policy.

## Substandard and Falsified Medicines in Africa – a hidden crisis

Substandard and falsified (SF) medicines are a pervasive but poorly mapped threat to health systems across Africa^1,2^. They undermine treatment efficacy, drive antimicrobial resistance, and cause preventable deaths, yet the scale of the problem remains uncertain^3,4^. SF medicines appear in both informal and formal supply chains—from street vendors to licensed pharmacies—but their relative contributions are rarely quantified^5,6^. This reflects not only weak regulation but also a deeper deficiency in the data and surveillance systems that should make poor-quality medicines visible.

By data and surveillance, we refer to the systematic collection, testing, and reporting of information on medicine quality—activities that enable regulators to identify, trace, and respond to SF products. In many African countries, such systems are fragmented, under-resourced, project-based, or externally driven, leaving significant gaps in national and regional coverage^7^. Without consistent, locally generated evidence, regulators operate with limited visibility, and policy responses risk being misdirected.

This Comment examines those evidence gaps directly. Drawing on data from the Medicine Quality Scientific Literature Surveyor, we show that research on SF medicines in Africa is heavily concentrated in a few countries and dominated by external institutions. The absence of coordinated, Africa-led research and surveillance infrastructure not only obscures the true scale of the problem but also constrains the continent’s ability to develop effective and sustainable responses.

## A Fragmented and Unequal Evidence Base

**Fig. 1:**
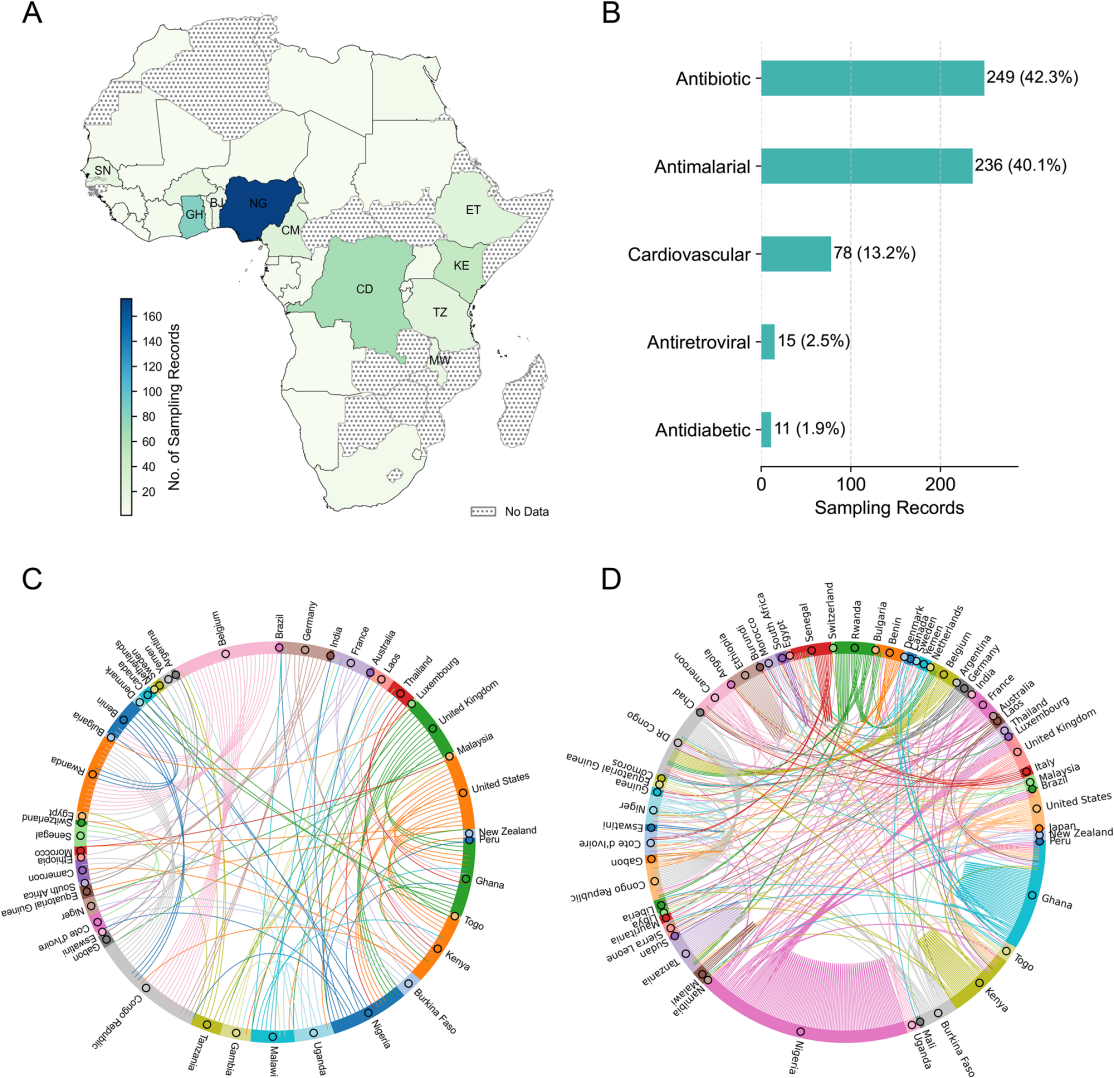
Mapping SF medicines research in Africa using data from Medicine Quality Scientific Literature Surveyor. The Surveyor is an online database developed by the Infectious Diseases Data Observatory (IDDO) and the Medicine Quality Research Group (MQRG)^8^, which aggregates and curates data from various sources, categorized by publication type as original research article, report, lay press, etc. We first identified 268 original research articles studying SF (either substandard or falsified or both) ‘Antibiotic’, Antidiabetic’, ‘Antimalarial’, ‘Antiretroviral’, and ‘Cardiovascular’ medicines worldwide between 1963 and 2021. **A** Out of these 268 original research articles, 150 articles consist of 589 sampling records of SF medicines in Africa. The distribution of these 589 sampling records on SF medicines is uneven, with the top ten countries being Nigeria (NG, *N* = 174, sampling ratio$\approx$30%), Ghana (GH, *N* = 83, sampling ratio$\approx$14%), Democratic Republic of the Congo (CD, *N* = 66, sampling ratio$\approx$11%), Kenya (KE, *N* = 49, sampling ratio$\approx$8%), Cameroon (CM, *N* = 26, sampling ratio$\approx$4%), Ethiopia (ET, *N* = 23, sampling ratio$\approx$4%), Tanzania (TZ, *N* = 20, sampling ratio$\approx$3%), Senegal (SN, *N* = 17, sampling ratio$\approx$3%), Benin (BJ, *N* = 15, sampling ratio$\approx$3%), and Malawi (MW, *N* = 14, sampling ratio$\approx$2%). **B** The counts of the five sampled categories of SF medicines in Africa show that most SF medicines sampling records within the 589 records are related to antibiotics and antimalarials. **C** Based on the 268 selected articles studying SF medicines worldwide, we constructed a collaboration network. This chord diagram indicates collaboration between countries whenever the authors’ institutions from different countries appear in the same article. Each node represents a country, with its size proportional to the number of unweighted edges connected to it (**D**). Notably, collaborations in the study of SF medicines were primarily composed of researchers in the Global North, working with researchers in just 23 African countries. While there were a few connections with countries in Asia and Latin America, intra-Africa collaboration on this issue was scarce, accounting for only 33 out of 141 edges (23.4%). **D** The selected 150 original research articles sampling SF medicines in Africa consisted of 185 unique sampling records containing information on authors’ countries and sampled African countries. This was used to construct an investigation network wherein the links indicate investigation directions originating from researchers’ countries to the sampled African countries. The per-country table for (**A**) and edge lists for (**C**, **D**), and the data collection procedure from the Surveyor can be found in the Supplementary Data and Code.

To grasp the extent of this evidence gap, we analysed 268 original research articles on SF antibiotics, antidiabetics, antimalarials, antiretrovirals, and cardiovascular medicines worldwide, published between 1963 and 2021 (as the dataset only goes up to this year at the time of access), using data from the Medicine Quality Scientific Literature Surveyor^8^. Just over 50 percent of the articles included African sampling records, and those records disproportionately focused on a few countries: Nigeria, Ghana, the Democratic Republic of Congo, and Kenya– with 19 out of 54 African countries lacking sampling records (Fig. 1). Most African countries are absent from the literature.

Moreover, as shown in Fig. 1, the types of medicines studied in Africa are narrowly clustered around antibiotics and antimalarials. Other drug classes, including those targeting non-communicable diseases, are significantly underrepresented. This uneven evidence base undermines the capacity of African regulators to make informed decisions. It hampers efforts to coordinate surveillance, target interventions, and evaluate the effectiveness of reforms.

Many investigations were conducted locally within the researchers’ home countries, as shown in Fig. 1D by the self-connected edges (128 out of 381 edges, 33.6%), predominantly in a few African countries. Notably, there were many investigations led by countries in the Global North. The figure reveals that research on SF medicines in Africa focused on a few African countries and was dominated by forces outside the continent, with the Global North playing the leading role, while intra-Africa collaboration remained underdeveloped.

Meanwhile, given the lack of data on post-2021 publications, the patterns of country and drug-class coverage in Fig. 1 might differ slightly, particularly due to COVID-19 pandemic impacts. Additionally, the dataset was primarily funded by Western funders and includes mainly English-language literature, potentially introducing bias by missing non-English and local language publications. This limitation highlights another inequality in public health database infrastructure in Africa.

## Patterns of Global Research Inequality

Our analyses reveal a stark imbalance in global research collaboration on SF medicines. Most studies involving SF medicines in Africa are led by researchers based in the Global North. While many include African collaborators, collaboration within Africa is minimal. Co-authorship networks uncover limited ties between African countries, and few studies are co-led or coordinated by African institutions that span borders (Fig. 1). This pattern is not unique to research on SF medicines; rather, it reflects a broader trend in Global North–South research collaboration, where Northern institutions often dominate agenda-setting, authorship, and leadership roles.

In the early 2000s, African governments pledged to allocate 1% of GDP to research and development (R&D), setting important policy benchmarks for advancing African-led research^9^. However, the implementation of this commitment has been uneven. Current data show that average R&D spending across the continent remains around 0.45% of GDP, with only a handful of countries approaching the 1% target^10^. Recent disruptions in external research funding highlight the urgency of revitalising this commitment to strengthen the continent’s research capacity and autonomy^11^.

## The Importance of Intra-African Research Collaboration

Strengthening intra-African research collaboration on SF medicines is essential for three reasons. First, locally grounded research enables context-sensitive detection and response. National regulators need data reflecting their specific pharmaceutical markets and border dynamics. Second, collaboration across African countries allows for the identification of transnational patterns in the circulation of SF medicines. Given porous borders and inter-country pharmaceutical trade, national approaches cannot succeed in isolation. Third, research collaboration fosters the development of local expertise and leadership. This is crucial not just for SF medicines but for broader health system resilience and pharmaceutical policy innovation.

## Building the Infrastructure of Collaboration

Efforts to combat SF medicines must include strategic investments by African countries in research and development ecosystems. Strengthening the continent’s scientific capacity is essential for developing locally grounded, sustainable solutions. We call for:

1. **Dedicated funding mechanisms** for Africa-led, cross-national research on medicine quality, ensuring that African institutions lead in shaping research agendas and their implementation. Within three years, at least three regional funding calls could be launched, with success measured by the proportion of projects led by African principal investigators.
2. **Incentives for South-South collaboration** that require co-leadership between institutions in different African countries for projects investigating transnational SF medicine flows, vulnerabilities in border markets, and comparative regulatory responses. A realistic target is a 25–30 percent increase in cross-country African co-authorships on studies of SF medicine within three years, reflecting stronger regional research networks.
3. **Support for open-access data platforms and regional knowledge-sharing hubs**, enabling real-time sharing of SF medicine sampling data, laboratory analyses, and regulatory alerts among national authorities and researchers. The African Medicines Agency could pilot a shared repository linking selected national medicine-quality laboratories.
4. **Integration of research functions within national regulatory agencies**, through the establishment of partnerships or memoranda of understanding with universities to embed research expertise within pharmacovigilance units. Progress could be measured by the presence of at least two such partnerships across different regions and the inclusion of research-derived evidence in annual regulatory reports within three years.
5. **Robust evaluation frameworks** that go beyond prevalence estimates to examine the social, economic, and supply chain conditions that enable the circulation of SF medicines. Within three to five years, coordinated comparative studies across at least five countries could establish common indicators—such as testing coverage, response times to SF medicine alerts, and regulatory follow-up rates—to track improvements in medicine quality and oversight capacity across the region.

The recent collaboration among African countries in combating the COVID-19 pandemic has established a paradigm for addressing complex health challenges on the continent^12^. Moreover, existing regional bodies—such as the East African Community (EAC), the Southern African Development Community (SADC), and the Economic Community of West African States (ECOWAS), together with the new African Medicines Agency (AMA), can foster practical, locally tailored frameworks for long-term collaboration. Only through a coordinated, Africa-centred approach that empowers local researchers and institutions can the continent build the evidence base necessary to confront the complex and evolving challenge of SF medicines.

## Conclusion

The fight against SF medicines is often framed as a regulatory challenge. Yet, it is equally a research challenge. Africa cannot regulate what it cannot see, and it cannot see what it has not studied. The current evidence base on SF medicines in Africa is too sparse, skewed, and externally driven to support meaningful and sustained progress. Addressing this requires a shift: from dependence on external studies to investment in Africa-based, Africa-led research collaborations. SF medicines are a global health threat. Ensuring they are properly studied, tracked, and defeated in Africa is not only an African priority but a global responsibility.

## Supplementary information

Description of Additional Supplementary Files

Supplementary Data and Code
