# Supplementary material for: The urgent need for African research collaboration on medicine quality
Source: Nat Commun. 2026 Jan 10;17:478. doi: 10.1038/s41467-025-67430-z (PMC12800327; doi:10.1038/s41467-025-67430-z)
Supplement: Supplementary file 1 — Description of Additional Supplementary Files [file 41467_2025_67430_MOESM1_ESM.pdf]

Description of Additional Supplementary Files

File Name: Supplementary Data and Code

Description: The per-country table for Fig. 1A and edge lists for Fig. 1C and 1D, and the data collection procedure from the Surveyor.
