# Supplementary material for: The urgent need for African research collaboration on medicine quality
Source: Nat Commun. 2026 Jan 10;17:478. doi: 10.1038/s41467-025-67430-z (PMC12800327; doi:10.1038/s41467-025-67430-z)
Supplement: Supplementary file 2 — Supplementary Data and Code [file 41467_2025_67430_MOESM2_ESM.zip › Supplementary Materials/Dataset Search and Collection Procedure.docx]

The steps **for searching for and collecting** the dataset used in the paper:

1. Go to the website of the Surveyor: <https://www.iddo.org/mq-scientific-literature-surveyor>
2. Click the 'View Map' button on the website, which will lead to another webpage: <https://www.iddo.org/mqsurveyor/>
3. **On** the new webpage, click 'Antimalarials' (there is no difference if **you** click other Medical Product Class names listed here for the following steps).
4. **On** the new webpage (<https://www.iddo.org/mqsurveyor/#antimalarials>), click **the** 'Download the data' button, **and** a zipped 'IDDO_MQ_Surveyor_Database.zip' file will **appear and can** be downloaded directly. The zip file contains all original data and some notes on the dataset.
5. In **this** work, we only select publications that belong to 'Original research article' under the 'publication_type_name' variable and **where the** medicine type **is** 'Antibiotic', **'Antidiabetic', 'Antimalarial', 'Antiretroviral', or 'Cardiovascular'. We then, based on** the 'sdq_category' variable, select substandard or falsified records. These steps **allowed us to obtain** the dataset we used in the paper.
6. In the original research article dataset, each row **represents** a sampling record, and each publication has a unique 'publication_id'. Please note that one **publication** may have multiple sampling records (rows).
7. Using this selected dataset, we **conducted the** analyses **presented** in the paper.
8. We accessed the Surveyor **on** 20 May 202**4**. **At the time of access**, the original data only extended to **2021**.
